# Supplementary material for: Flagellar Kinematics and Swimming of Algal Cells in Viscoelastic Fluids
Source: Sci Rep. 2015 Mar 17;5:9190. doi: 10.1038/srep09190 (PMC4361863; doi:10.1038/srep09190)
Supplement: Supplementary Information — SI - Text and Figures [file srep09190-s1.pdf]

Supplementary Information: Flagellar Kinematics and Swimming of Algal cells in Viscoelastic Fluids

B. Qin,[1] A. Gopinath,[2,1] J. Yang,[2,1] J. P. Gollub,[2] and P. E. Arratia[3,a)]

[1] *Department of Mechanical Engineering & Applied Mechanics, University of Pennsylvania, Philadelphia, PA 19104*

[2] *Department of Physics & Astronomy, Haverford College, Haverford, PA 19041*

[3] *Department of Mechanical Engineering & Applied Mechanics, University of Pennsylvania, Philadelphia, PA 19104*

(Dated: 4 February 2015)

[a)] Author to whom correspondence should be addressed. Electronic mail: parratia@seas.upenn.edu.

I. FLUID RHEOLOGY

Fluids are characterized using a stress-controlled cone-and-plate rheometer (RSF 3, TA Instruments). All measurements are performed at constant temperature, 23°C.

A. Shear viscosity

Two types of fluids are used: Newtonian and viscoelastic. Newtonian fluids are prepared by dissolving relatively small quantities of Ficoll (Sigma-Aldrich) in M1 buffer solution. The Ficoll concentration in M1 buffer ranged from 5% to 20% by weight. Figure 1 shows the shear-viscosity as a function of shear rate for several Ficoll solutions. We find nearly constant viscosity behavior even for a very high Ficoll concentration (40%) solution, indicating that shear-thinning behavior is negligible.

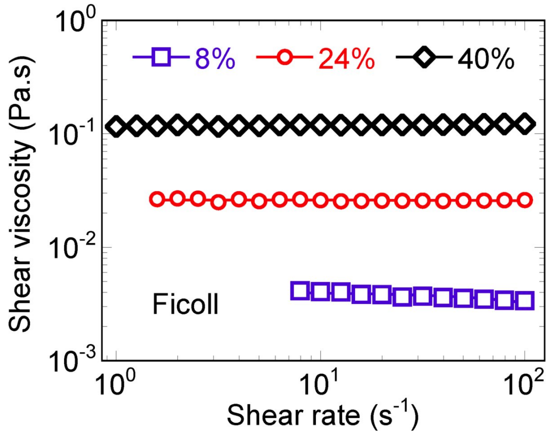

FIG. 1. Ficoll solutions viscosity versus shear rate data for 8%, 24% and 40% (by wt) polymer concentration.

Viscoelastic fluids are prepared by adding small amounts of the high molecular weight, flexible polymer polyacrylamide (PAA, MW $= 18 \times 10^6$) to water. The concentration of PAA polymer in water ranged from 5 to 80 ppm; PAA overlap concentration is 350 ppm. Sample flow curves for PAA solutions are presented in Fig. 2. We find nearly constant viscosity for all fluids except for the highest concentration case of 80 ppm, where shear-thinning viscosity is observed. At the most shear thinning case, namely the 80ppm PPA concentration, the shear rate of the algae body $\dot{\gamma}_{\text{body}}$, which sets the lower bound of the $\dot{\gamma}$, can be estimated by $\dot{\gamma}_{body} = |U|/D \approx 15s^{-1}$, where $|U|$ is the average of the power and recovery stroke speeds and D is the cell diameter. With this estimate, the cell body of average algae experience a

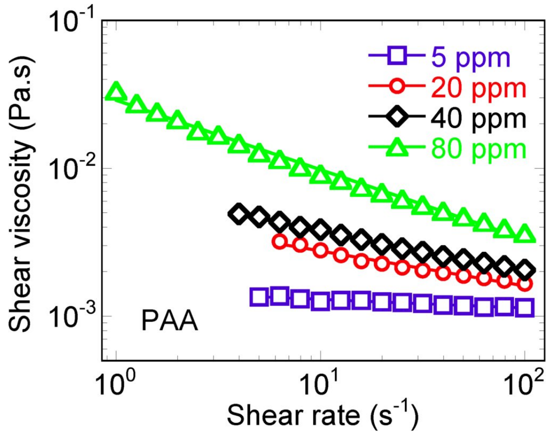

FIG. 2. Shear viscosity curves, $\eta(\dot{\gamma})$ as a function of shear rate $\dot{\gamma}$ of PAA solutions for concentrations ranging from 5 ppm to 80 ppm.

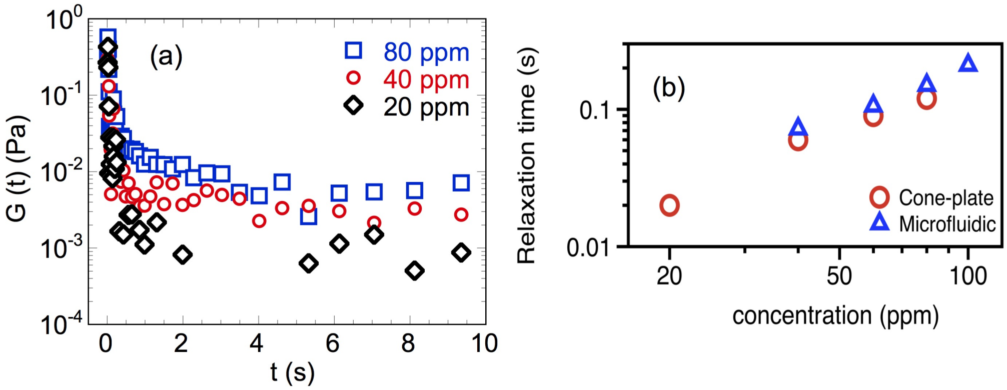

FIG. 3. (a) Sample stress relaxation curves for PAA solutions with concentrations ranging from 20 ppm to 80 ppm. (b) Comparison of relaxation times obtained using commercially-available rheometer (RSF3, TA Instrument) and an in-house developed microfluidic rheometer[1]. Values are very similar.

shear viscosity of $\eta_{\text{body}} \approx 6.5$ mPa·s. On the other hand, the shear rate experienced by the flagellum can be estimated by the flagellar beating frequency $\dot{\gamma}_{\text{flag}} \approx 50s^{-1}$ and consequently the shear viscosity experience by the flagella is $\eta_{\text{flag}} \approx 5$mPa·s, for the most shear thinning case. The viscosity used to report our kinematic data is taken to be the shear viscosity at the mean of the two shear rate. The deviation from this mean estimate is 13% at the most concentrated fluid.

PAA (ppm)	λ (s)
5	0.006*
20	0.02
40	0.06
60	0.09
80	0.12

TABLE I. Relaxation times of PAA solutions. The superscript * indicates that λ was obtained via extrapolation.

.

B. Fluid relaxation times

Fluid relaxation times λ are obtained by fitting the stress relaxation data with the generalized linear viscoelastic model of a single relaxation time of the type $G(t) = G_0 e^{-t/\lambda}$, where $G(t)$ is the fluid shear modulus and λ is the fluid relaxation time. Figure 3 (a) shows sample curves of stress relaxation data. The relaxation times of PAA fluids are given in Table 1. Due to the limits in instrument sensitivity and accuracy, the fluid relaxation time for lowest concentration case (5 ppm) is obtained by extrapolating the experimental data. We also measured the values of λ using a newly-developed microfluidic rheometer[1] capable of measuring fluid relaxations down to 10 ms. Figure 3(b) shows a comparison between the values of λ obtained using the RF3 and the microfluidic rheometers. The values are quite similar, and that give us confidence in our measurements of λ.

II. TRACKING AND IMAGE ANALYSIS

A. Swimming (signed) Speed

Standard particle tracking techniques are used to locate the centroid of the cell body. Raw images of selected swimming alga first undergo image processing including contrast enhancement and band pass filtering for the removal of the high-frequency noise in the image. The centroid of the cell body for each image is then obtained using a non-linear least square method. The magnitude of the instantaneous swimming speed (or signed speed) is the ratio

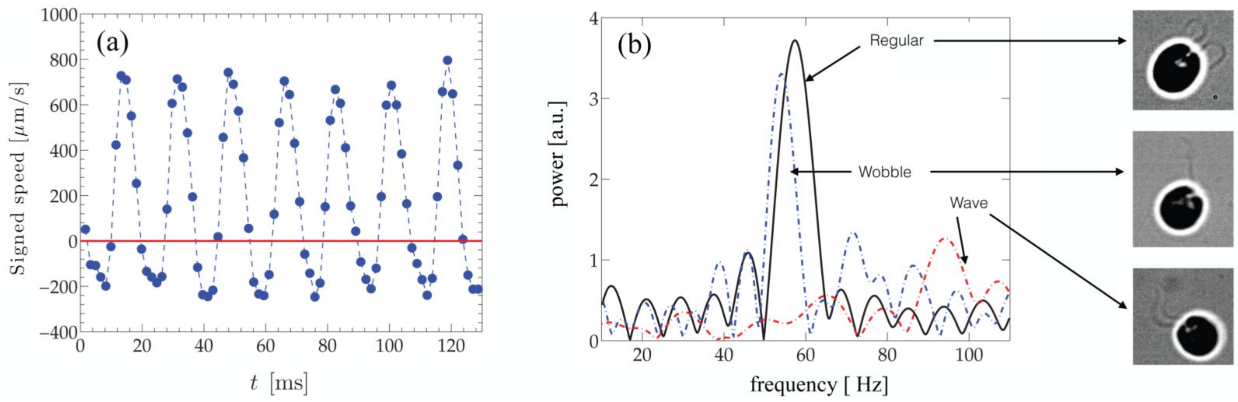

FIG. 4. (Color online) (a) The (signed) speed of the cell body over consecutive cycles. We notice the forward (speed > 0) and backward (speed < 0) motion are observed during the power and recovery stroke respectively. (b) Frequency spectrum of cell body speed for three different swimming gaits present in our experiments at a viscosity of $\mu = 1$ mPa·s. Regular strokes (black curve), wherein both flagella beat symmetrically and at the same frequency, results in a single distinct peak of around 60 Hz for this particular alga. We also observe "wobbles" (dashed blue curve) where the two flagella are out-of-sync and beat irregularly, illustrated by a second peak at higher frequency $\sim$ 70 Hz. A third swimming mode was seen - we term this the wave - where the alga send synchronous traveling waves down each flagellum (dashed red curve). In this mode, the peak power spectrum of the body speed is much weaker and occurs at very high frequency $\sim$ 95 Hz. Snapshots of the different beating modes are denoted in the panels on the right (Videos are provided as online supplementary materials - see SI-Videos).

of the magnitude of the displacement vector over the time step. The sign of the instantaneous speed, denoting forward (+) or backward (-) motion, is determined by inspecting the angle between the displacement vector and the cell body orientation vector, which is from cell centroid to the cell head. The power stroke mean speed and recovery stroke mean speed can obtained by averaging the signed speed in the respective stroke over multiple cycles. The net speed is then the averaged over both power and recovery strokes.

Care was taken to ensure that the algae being tracked are swimming with the "regular" stroke pattern wherein the two flagella are both actively beating with matching frequency. Experimentally, such regular stroke patterns are evidenced in a clear singular peak in the frequency spectrum of the instantaneous speed cf. Figure 4(b). In contrast, wobbling cells,

whose two flagella beat with different frequency, have a second peak at a higher frequency. Finally, cells whose flagella propagate synchronous traveling waves and dead cells have peaks at unusually high frequency and energy spectra that are more spread-out. Videos of these swimming modes are in online supplementary materials. For each rheologically distinct fluid, 10-20 sample individuals are obtained whose speed spectrums satisfy our requirement.

B. Flagellar Beating Contours

Material points along the arc-length of each flagella over many complete beating cycles are tracked using pixel-intensity-weighted averaging and iteration based on resultant centerline curvature. The contour (shape) was manually identified in the image and the pixel positions and intensities corresponding to the material points on the flagella are extracted - c.f Figure 5(a). These points (~ 200) are then grouped into 32 subsets based on their mutual proximity to obtain 32 centerline loci of the flagellum. A cut-off radius is set equal to the flagellar radius and was used for the grouping. The first approximation to the positions is obtained by finding the centroid of the subsets with the weight set to pixel intensity. The resulting positions of the centerline points are then smoothed by fitting a cubic spline that minimizes least-square error and second derivative ("curvature") with a weight of 4:1. Using new position estimates, the procedure is repeated until the filament curvature calculated from the x-y positions varies little between iterations (<1%). Finally, we record the centerline position relative to the centroid of the cell body and interpolate to obtain evenly spaced points. A pictorial summary of this process is outline in Figure 5(b). This process reduces the error due optical noise under high magnifications.

Once sequential flagellar contours within a beating cycle are obtained, results for multiple periods (typically 6-8) are then mapped (folded) in time and assigned a dimensionless phase ratio parameter ϕ, assuming constant beating frequency. The locus of centerline x-y positions, as functions of phase ratio ϕ and arc length s, are then smoothed by fitting a surface that minimizes least-square error and second derivatives (surface "curvature"). A typical folded contour obtained in this manner and the raw sampled contour positions are compared in Figure 5(c), by tracking the tip of the flagellum at 8μm from the proximal

end. Agreement is seen with the raw centerline points. The filament contours during power stroke and recovery stroke are obtained in accordance to the convention that power stroke is when the cell body moves with a component forward while recovery stroke is when the cell body moves backward.

The sampled individuals were carefully checked to ensure that both the cell body and the flagella remain in focus throughout the sampling period of the flagellar waveform. Individuals that display a "merging" of the flagellar signal (indicating rotating about the orientation axis) and whose bodies defocus are not included in the statistics. At our current imaging frame rate (600 Hz), the noise to signal ratio at the last 5-10% of the flagella is often relatively greater due to the higher speed at the distal end. Thus we have excluded this region of low confidence. Finally we note the effective flagellar length, the length up to which flagellar kinematic data are reliable, is fairly constant, $\ell \approx 8$ μm, for all fluids and individual cases serving as a check for the consistency of the image analysis.

Since there can still be some variability due to individual differences, we checked that flagella contours obtained were indeed representative of a population. The data in Figure 5(d) shows point clouds at four different locations along the flagellum (4 values of s/ℓ) for a single fluid experiment (80 ppm, De = 6.5) presented in Figure 1 of the main text. The data points are positions obtained from image analysis of 3 separate individuals, each over 5 beating periods. For each of the four values of s/ℓ, the points collapse quite well indicating that variations between specimens are not strong compared to the mean. Note that time is an intrinsic parameter and the overlap between clouds is only apparent and not real.

Finally, the comparison of the flagellar waveform and curvature kymographs between the Newtonian and the viscoelastic case at $\mu \approx 2.6$mPa·s, De=2.4 is shown in Figure 7, as supplement to the analogous comparison made at $\mu \approx 6$mPa·s in Figure 1 of the main text.

III. BEAT FREQUENCY

The flagella, at a coarse grained level, constitute an active Euler beam of high slenderness ratio (i.e, filament) driven internally by the sliding of molecular motors. Current theories suggest that constitutes an emergent self-organized beating micro-machine. Theoretical models for the onset of ciliary/flagellar beating and the frequency of emergent oscillations models[2,3] suggest that the frequency is selected based on a linear stability constraint - this frequency changes well into the non-linear regime that corresponds to the oscillations we seen in the experiment. The beating amplitude and its saturation is purely dictated by non-linearities, both geometric as well as kinetic.

In the following, we adapt these models to obtain a simple relationship between the beating frequency and the external fluid viscosity. While these simple models assume a base state that is straight and do not include the possibility of different power and recovery strokes the physical picture relating the internal driving activity to observed oscillation frequency is the same and thus we expect to at least get qualitative information.

A cilium or a flagellum beating in a liquid of viscosity μ is modeled as two slender filaments (radius a, length ℓ and bending modulus B_{pas}) sliding relative to one another due to the action of active dynein motors. The internal passive links - such as nexin links - are assumed to yield an effective elastic resistance corresponding to a linear spring and are coarse-grained into an internal spring constant per unit length k_{N}. This forced sliding combined with the geometric constraint at the base and the bending and shear resistances (due to nexins) yields bending. Depending on parameters and boundary conditions, the sliding can be oscillatory in nature and thereby generate propagating bending waves.

For small deformations valid close to onset of oscillations, the lateral displacement measured as a function of arc-length h is related to the local curvature κ by its second derivative w.r.t arc-length - i.e, $\kappa = h''$. Using $h = \mathrm{Real}[\overline{h}e^{i\omega t}]$ where ω is the emergent frequency and noting that for *C. reinhardtii* we may approximate the boundary conditions to be clamped at the basal end and free at the distal end, that is $\overline{h}(s=0) = \overline{h}'(s=0) = 0$ and

$\overline{h}''(s=\ell) = \overline{h}'''(s=\ell) = 0$. The equation for $\overline{h}$ is to linear order,

$$\mu i\omega\overline{h} + \left(B_{\mathrm{pas}}\overline{h}'' - a^2 k_{\mathrm{N}}\overline{h}\right)'' = a^2\chi\overline{h}''$$

where the active, frequency compliance $\chi(\omega)$ arises from the viscoelastic nature of the motor activity - akin to an active viscoelasticity of the material inside the flagellum. Note that in the absence of activity solutions are rapidly damped down by viscosity over a elasto-viscous time scale $\tau_{\mathrm{ev}} \equiv \omega_{\mathrm{ev}}^{-1} \sim \mu\ell^4/B_{\mathrm{pas}}$. Since the frequencies are not linearly decaying with viscosity, the beating is driven actively and does not correspond to passive relaxation.

Solutions are possible only for special value of ω - these thus constitute eigenvalues at which oscillatory solutions arise. We choose a simple expression for $\chi(\omega)$ from Camalet and Julicher[2] that involves an kinetic rate (ATP cycling rate) α_{ATP} and simplify it for the case at hand. The most unstable mode is associated with oscillations that emerge with frequency

$$\omega \sim \sqrt{\left(\frac{B_{\mathrm{pas}}}{\mu\ell^4}\,\alpha_{\mathrm{ATP}}\right)}\left(1 + \frac{4k_{\mathrm{N}}\ell^2 a^2}{\pi B_{\mathrm{pas}}}\right)$$

thus yielding the scaling $\omega \sim 1/\sqrt{\mu}$. It is intriguing to see that our experiments - c.f Figure 2(a) in the main text - seem to be consistent with this prediction.

We would like to point out that the presence of the nexin links results in an effectively larger bending modulus due to shear stiffening - that is we can identify an effective bending stiffness (passive) $B_{\mathrm{eff}} \sim B_{\mathrm{pas}}\left(1 + 4k_{\mathrm{N}}\ell^2 a^2/\pi B_{\mathrm{pas}}\right)^2$ due to the shear stiffening of the springs. Finally we also note that this expression assumes that there is no passive viscosity inside the flagella. In reality the filaments are affected by external (μ) and internal (μ_{int}) viscosities and so a rough argument would suggest replacing μ by an effective viscosity $\mu_{\mathrm{eff}} \sim \mu + \mu_{\mathrm{int}}$. When $\mu \gg \mu_{\mathrm{int}}$, we find $\omega \sim 1/\sqrt{\mu}$ while when $\mu \ll \mu_{\mathrm{int}}$, we expect $\omega \sim 1/\sqrt{\mu_{\mathrm{int}}}$ - a constant dependent on the internal dissipative friction. This is consistent with our observed frequencies as for low viscosities the frequency seems to tend to a constant.

REFERENCES

[1]Koser, A. E., Pan, L., Keim, N. C. & Arratia, P.E., *Lab Chip*, 13, 1850 (2013).

[2]Camalet, S & Jülicher, F., *New J. Phys.*, 2, 24.1 (2000).

[3]Grill, S. W., Kruse, K., & Jülicher, F., *Phys. Rev. Lett.*, 94, 108104 (2005).

SI: MOVIE CAPTIONS

SIRegular

Video showing regular beating in an alga - this is the swimming gait used in the analysis. The two flagella beat synchronously (with mirror symmetry about the centerline) using clear power and recovery strokes. During the power stroke, the cell body moves forward; during the recovery stroke it move backward. The power and frequency spectra show a clear peak.

SIWobble

Video showing wobble in an alga. The two flagella beat using the regular gait - except that every now and then, the trajectory is punctuated by a wobble where one flagellum goes out of synchrony with the other. These wobbles are analogous to tumbles in prokaryotic bacteria and may correspond to turning events.

SIWave

Video showing wave like beating in an alga. The two flagella beat in the same direction (no mirror symmetry about the centerline) by propagating bending waves along the flagellum. While this does lead to locomotion, the speeds we observe are not high - besides, this is not the usual swimming gait used.

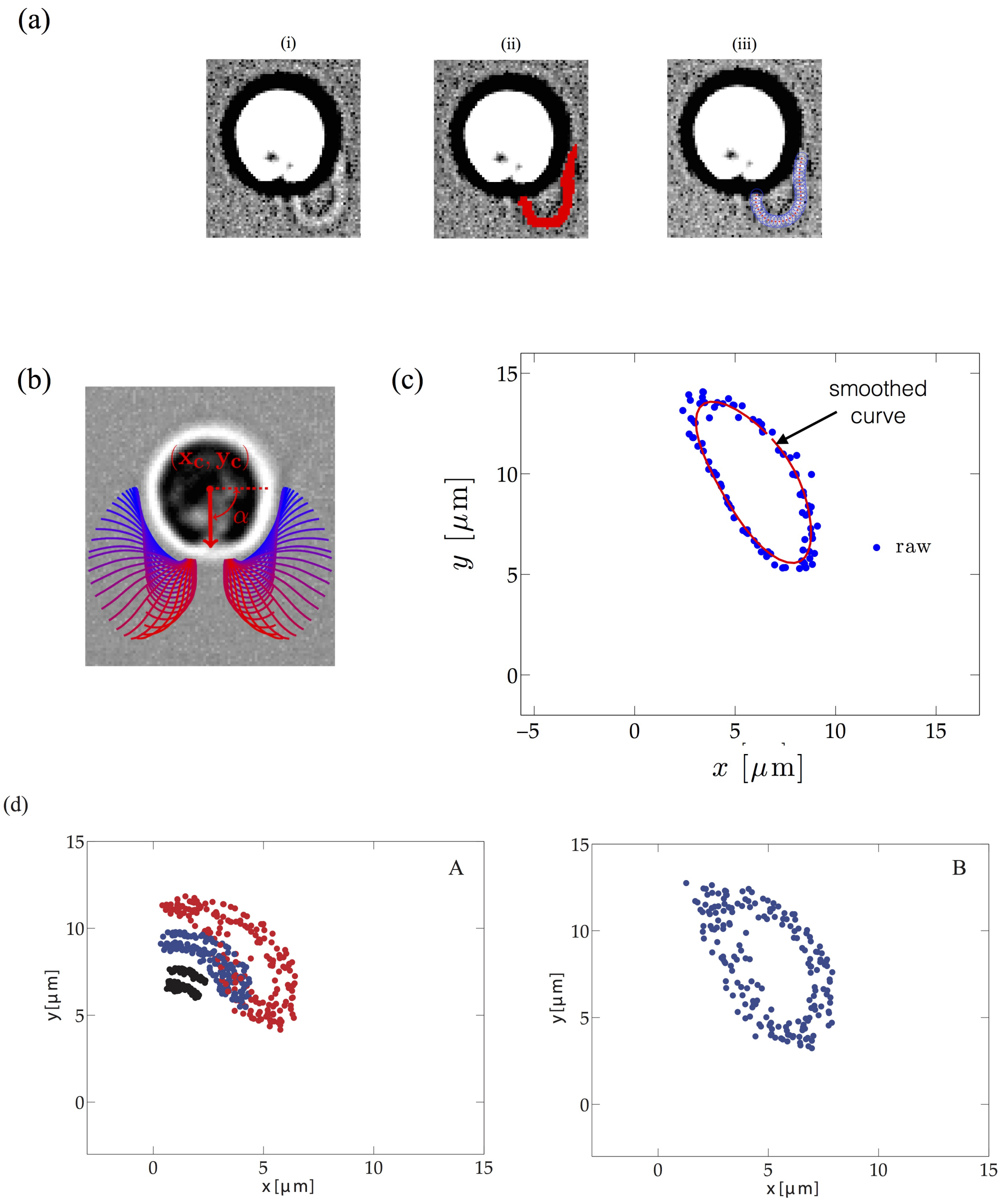

FIG. 5. (color online) (a) Sequence of steps (i)-(iii) in extracting flagellar centerline positions and generating continuous differentiable contours. (b) Final contours illustrating the symmetry of the beat, the position of the centroid (x_c, y_c) and cell orientation α. (c) The comparison of the raw sampled centerline points at $s = 8\mu$m from the proximal end over time versus those after the smoothing in phase, ϕ and arc-length s. We see that the noise level is low and that such smoothed loci capture the actual tip positions faithfully. (d) To demonstrate that the captured flagellar waveform is indeed representative of the sampled population, we plot the trajectories of the flagellum at fixed positions $s/\ell = 0.2$(A, black), 0.5(A, blue), 0.8 (A,red), 1 (B, blue), for a total of 3 individuals each over 5 beating periods. The fluid in which the samples are observed is 80ppm PAA solution.

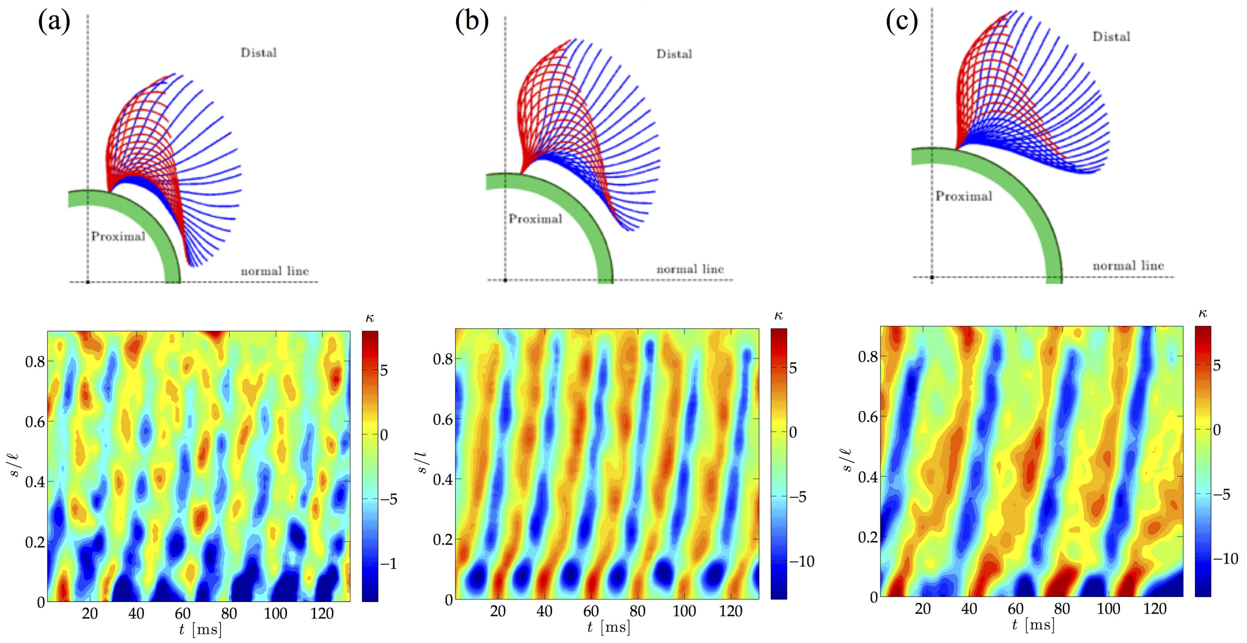

FIG. 6. (color online) Typical flagellar shape contours corresponding kymographs for purely Newtonian fluids at three different viscosities (a) 1 cP, (b) 2.6 cP and (c) 6 cP.

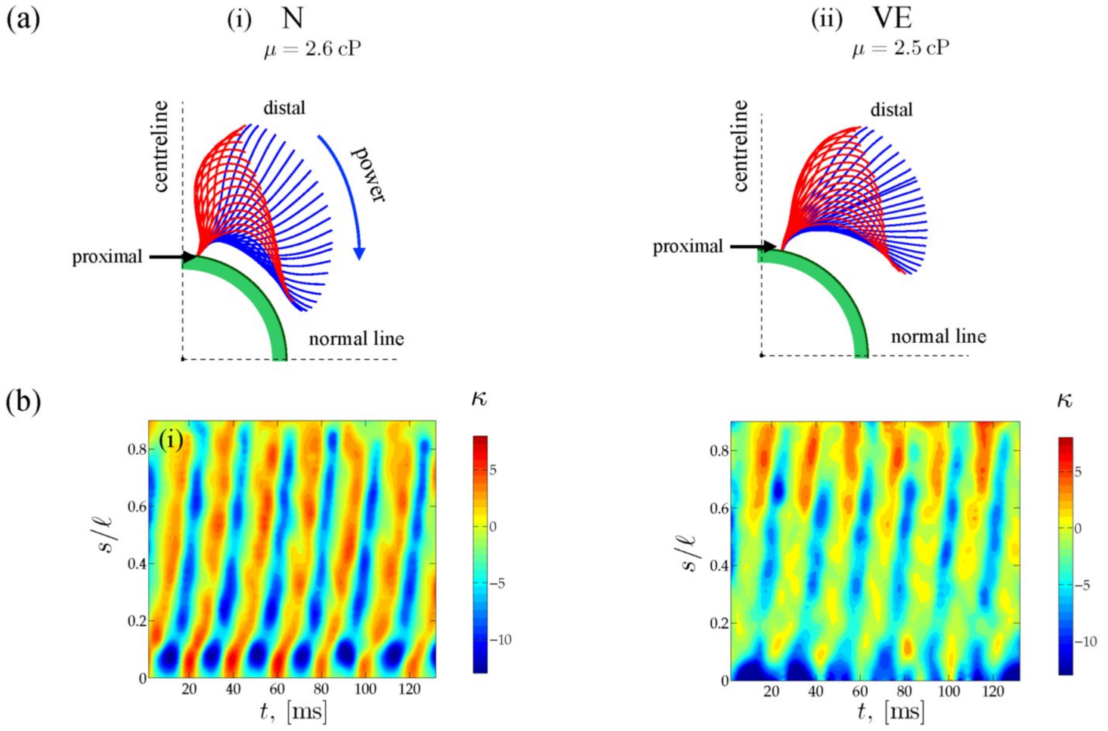

FIG. 7. (color online) (a) Flagellar shape contours at $\mu \approx 2.6$mPa·s (arrows indicate direction of beating) for (i) Newtonian and (ii) polymeric fluid De=2.4. (b) Corresponding spatio-temporal normalized curvature distributions along the flagella in Newtonian (left panel) and viscoelastic fluid (right panel).
